# Collagen XXV promotes myoblast fusion during myogenic differentiation and muscle formation

**DOI:** 10.1038/s41598-019-42296-6

**Published:** 2019-04-10

**Authors:** Tristan J. M. Gonçalves, Florence Boutillon, Suzie Lefebvre, Vincent Goffin, Takeshi Iwatsubo, Tomoko Wakabayashi, Franck Oury, Anne-Sophie Armand

**Affiliations:** 1 INSERM UMRS 1124, 45 rue des Saints-Pères, F-75270 Paris cedex 06, France; 2 Institut Necker-Enfants Malades, Inserm, U1151, 14 rue Maria Helena Vieira Da Silva, CS 61431, Paris, F-75014 France; 3 Université Paris Descartes, Sorbonne Paris Cité, Paris, France; 4 Department of Neuropathology, Graduate School of Medicine, the University of Tokyo, Tokyo, 113-0033 Japan; 5 Department of Innovative Dementia Prevention, Graduate School of Medicine, the University of Tokyo, Tokyo, 113-0033 Japan

**Keywords:** Extracellular signalling molecules, Differentiation, Musculoskeletal development, miRNAs

## Abstract

Fusion of myoblasts into multinucleated myofibers is crucial for skeletal muscle development and regeneration. However, the mechanisms controlling this process remain to be determined. Here we identified the involvement of a new extracellular matrix protein in myoblast fusion. Collagen XXV is a transmembrane-type collagen highly transcribed during early myogenesis when primary myofibers form. Limb muscles of E12.5 and E14.5 *Col25a1*−/− embryos show a clear defect in the formation of multinucleated myofibers. In cell culture, the cleaved soluble extracellular domain of the collagen XXV is sufficient to promote the formation of highly multinucleated myofibers. *Col25a1* is transiently expressed during myogenic differentiation and *Col25a1* transcripts are down-regulated in multinucleated myofibers by a muscle-specific microRNA, miR-499. Altogether, these findings indicate that collagen XXV is required *in vivo* and *in vitro* for the fusion of myoblasts into myofibers and give further evidence that microRNAs participate to the regulation of this process.

## Introduction

Skeletal muscle development and growth is a highly regulated process controlled by interactions between muscle cells and their surrounding microenvironment. Skeletal muscle formation can be divided into several sequential events, as migration of muscle precursor cells (mpc), proliferation of myoblasts, cell cycle arrest, myoblast differentiation and fusion. These different steps are characterized by the expression of muscle specific transcription factors, the muscle regulatory factors (MRFs), Myf5, MyoD, myogenin and MRF4. Myf5 and MyoD promote expansion of mpc, whereas myoblast differentiation is driven by myogenin expression. During mouse development, trunk and limb muscles arise from two waves of myogenesis involving different populations of myoblasts^1^. Between E11 and E14.5, embryonic (primary) myoblasts align and fuse to form small multinucleated primary myofibers. From E14.5 to birth, fetal (secondary) myoblasts enter the myogenic program, adhere to the primary fibers, use them as a scaffold and fuse together to generate secondary myotubes^1,2^.

The different steps of myogenesis are linked to remodeling of extracellular matrix (ECM) proteins as well as by changes in the expression pattern of several cell surface receptors, such as integrins^3^. Mpcs are able to regulate their extracellular environment in adult myogenesis, by secreting microRNAs targeting synthesis of collagen by surrounding fibroblasts^4^. Collagens are major ECM proteins involved in different functions, from the formation of fibrillar networks of the ECM to cell adhesion and have been linked to muscular dystrophies^5,6^. Among the collagen family, type XXV collagen is highly expressed in developing limb skeletal muscles and in differentiating C2C12 myotubes, suggesting a role in the myogenic differentiation^7^. This collagen belongs to the MACIT (membrane-associated collagens with interrupted triple helices) subset of the collagen superfamily, together with type XIII, XVII and XXIII collagens^8^. This subfamily of collagen is suspected to be involved in cell adhesion. Collagen XVII, also called BP180, is a well-characterized structural component of hemidesmosomes, anchoring epidermal keratinocytes to the underlying basement membranes^9^. The other MACIT members are structurally different from collagen XVII, each with four noncollagenous and three collagenous domains, a short intracellular region and a furin-cleavage site^10,11^. Collagen XXIII is mainly expressed in epidermis and epithelia^12^ and likely mediates metastasis by facilitating cell-cell and cell-matrix adhesion^13^. Type XIII collagen probably functions as an adhesion molecule, since it has been localized at cell-cell and cell-matrix junction sites^14^. It has also been shown to be part of the neuromuscular junctions (NMJ) and essential for the correct assembly of various components of NMJ during the post-natal period in mice^15,16^.

Collagen XXV was first described as a component of senile plaques in Alzheimer’s disease brain^10^. The ectodomain fragment obtained after cleavage by furin convertase was called CLAC (collagenous Alzheimer amyloid plaque component) and transmembrane collagen XXV as the precursor of CLAC (CLAC-P). *Col25a1* knock out mice die at birth due to respiratory defects. Motoneurons fail to branch the diaphragm muscle and subsequently degenerate, indicating that this transmembrane collagen is probably required for proper muscle innervation during fetal mouse development^7^. However, *Col25a1* transcripts were detected in differentiating limb myofibers from early E11.5, long before muscle innervation, suggesting that this collagen could also be involved in early myogenesis steps.

In this study, we report the involvement of collagen XXV in early myogenesis. *Col25a1*−/− mice exhibit a delay in the fusion and organization of myofibers during primary myogenesis. In culture, *Col25a1* transcripts are transiently expressed during myogenic differentiation. The cleaved soluble ectodomain of collagen XXV is sufficient to facilitate fusion of myoblasts into myotubes, confirming the role of collagen XXV in myoblast fusion. As myotubes mature, a muscle specific microRNA, miR-499, represses Col25a1 expression, by targeting its transcripts. Collectively, our data reveal a new regulator of fusion of myoblasts into myotubes, collagen XXV, and highlight the involvement of muscle specific microRNAs in this process.

## Results

### Collagen XXV is required for muscle formation *in vivo*

**Figure 1:**
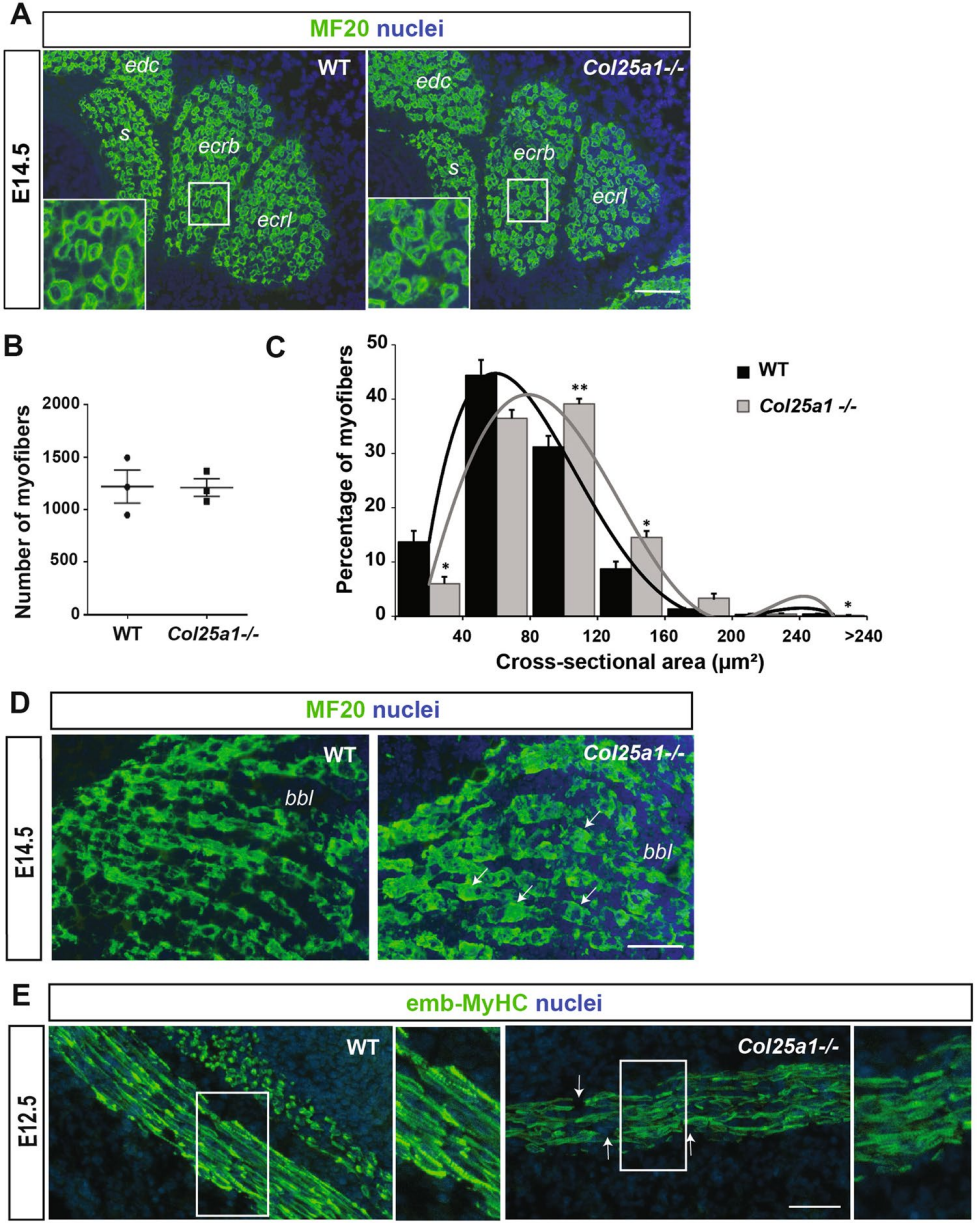
*Col25a1* expression is required for muscle development in mice. (**A**) Immunofluorescence analysis of myosin positive myofibers on transverse sections of forelimbs from wild-type (WT) and *Col25a1*−/− E14.5 embryos. Total nuclei were stained with Hoechst. Scale bar: 30 µm. *edc: extensor digitorum communis; s: supinator; ecrb: extensor carpi radialis brevis; ecrl: extensor carpi radialis longus*. (**B**) Quantitation of myosin positive myofibers on transverse sections of forelimbs from E14.5 WT and *Col25a1*−/− embryos. (**C**) Distribution of myosin positive myofiber cross-sectional areas (n = 3 animals of each genotype). *P < 0.05; **P < 0.01 vs WT. (**D**) Myosin immunofluorescence on forelimb longitudinal muscles from WT and *Col25a1*−/− E14.5 embryos (n = 3). Multinucleated myofibers were observed in WT sections. *Col25a1*−/− myofibers were myosin positive but shorter. Scale bar: 50 µm. Arrows indicate bulges. *bbl: biceps brachii long head*. (**E**) E12.5 forelimbs (n = 4) were immunostained with an antibody directed against the embryonic isoform of MyHC (emb-MyHC). *Col25a1*−/− myoblasts fusion seems to be delayed. High magnification of emb-MyHC staining (right) corresponds to area in white box from the left panel. Scale bar: 50 µm. Arrows indicate breaks between aligned myofibers.

Previous studies indicated that *Col25a1* is highly transcribed in developing skeletal muscle cells in limbs from E11.5 mouse embryos and not detected in adult skeletal muscles^7,10^. To examine the early *in vivo* function of collagen XXV, we analyzed the muscle phenotype of limb muscles from *Col25a1* knock out mice at E12.5 and E14.5 (Fig. 1). Histological analysis of transverse cross-sections of hindlimb muscles revealed the presence of myosin positive muscle cells in E14.5 *Col25a1*−/− mouse embryos (Fig. 1A). Their number is similar in *Col25a1*−/− and wild-type (WT) E14.5 embryos (Fig. 1B). However, muscle fibers of *Col25a1*−/− embryos are larger than controls and irregular in shape, whereas WT myofibers are linear (Fig. 1C). At this embryonic stage, the proportion of myofibers with a cross-sectional area above 80 µm^2^ is much higher in the *Col25a1*−/− forelimb muscles than in the same muscle masses in the control embryos. While multinucleated myofibers were present in forelimbs from WT E14.5 embryos, many of the myosin positive longitudinal myofibers observed in the *Col25a1* deficient forelimbs were much shorter, with few myonuclei (Fig. 1D). Some of them have bulges, which could explain their larger diameter compared to controls (Fig. 1C,D). Analysis of forelimbs from E12.5 WT and *Col25a1*−/− embryos strongly suggests that collagen XXV is required for the formation of multinucleated myofibers during primary myogenesis (Fig. 1E). Multinucleated myofibers with sarcomeres were detected in the control E12.5 embryos, whereas most of the *Col25a1*−/− myofibers seem to properly align but were much shorter. This suggests that collagen XXV expression is important for myoblasts to properly fuse, although we did detect multinucleated myofibers at later embryonic stages. As WT myofibers, *Col25a1*−/− fibers had sarcomeres and expressed embryonic MyHC. The diameter of these poorly nucleated myofibers seem to be larger, as already observed in forelimbs from E14.5 embryos (Fig. 1C–E). These *in vivo* data reveal that collagen XXV is required for muscle formation during primary myogenesis.

### Col25a1 is transiently expressed during myogenic differentiation

**Figure 2:**
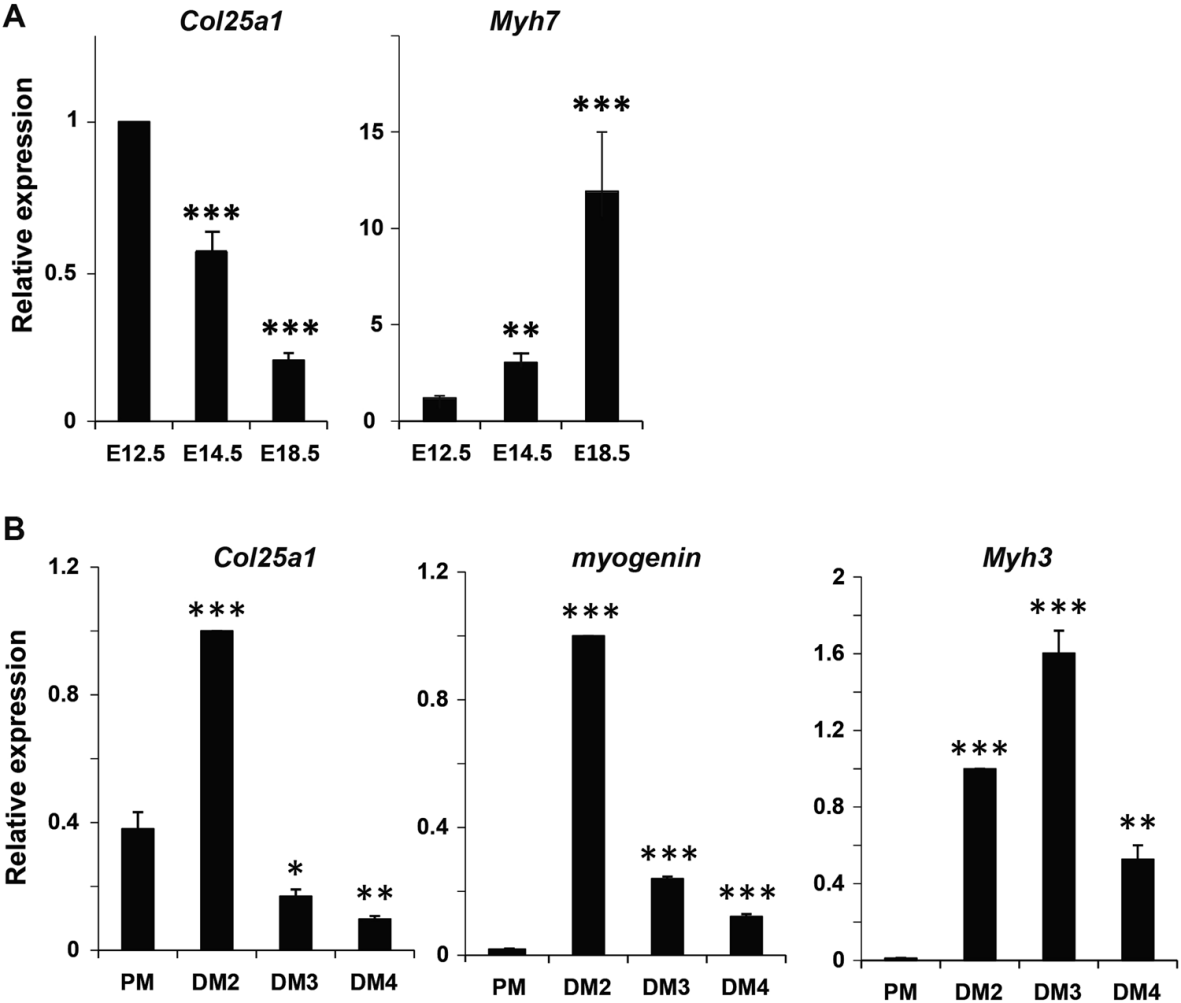
*Col25a1* expression during myogenic differentiation. (**A**) The expression of *Col25a1* and *Myh7* was analyzed by RT-qPCR from E12.5, E14.5 WT limb buds and E18.5 WT hindlimb muscles. Data are means ± s.e.m of three independent experiments. Three to six embryos were analyzed in each experiment. ** indicates P < 0.01; ***P < 0.001 vs E12.5 limbs. (**B**) Juvenile satellite cells were isolated from WT 10 day old mice and expanded. The expression profile of *Col25a1*, *myogenin* and *Myh3* was analyzed by RT-qPCR in cells cultured in proliferation medium (PM), or 2 (DM2), 3 (DM3) or 4 (DM4) days in differentiation medium. Data are means ± s.e.m of three different experiments. * indicates P < 0.05; **P < 0.01; ***P < 0.001 vs PM.

To better characterize the role of collagen XXV during primary myogenesis, as well as in myogenic differentiation, we analyzed the precise timing of *Col25a1* expression during mouse development and in cell culture. By RT-qPCR, we confirmed that *Col25a1* transcripts were abundant in limbs from WT E12.5 embryos and decreased when skeletal muscles mature, as measured by the abundance of *Myh7* transcripts, coding for the adult slow isoform of myosin heavy chain (type I MyHC) (Fig. 2A)^7^. Next, we analyzed the precise timing of *Col25a1* induction upon myogenic differentiation (Fig. 2B). Primary myoblasts were isolated from P10 neonatal WT mice and induced to differentiate. RT-qPCR analyses revealed a massive and transient induction of *Col25a1* transcripts at two days of differentiation, a time point which corresponds with the peak of expression of the differentiation marker, myogenin, and the initiation of transcription of the embryonic isoform of MyHC, encoded by the *Myh3* gene, specifically expressed in young myotubes. These results should be confirmed at the protein levels. Unfortunately, we were not able to detect the endogenous protein, since all antibodies tested were not specific enough. Altogether, our transcriptional expression results of *col25a1* during myogenic differentiation strongly suggest that collagen XXV is involved in the formation of myotubes.

### Collagen XXV promotes fusion of myoblasts

**Figure 3:**
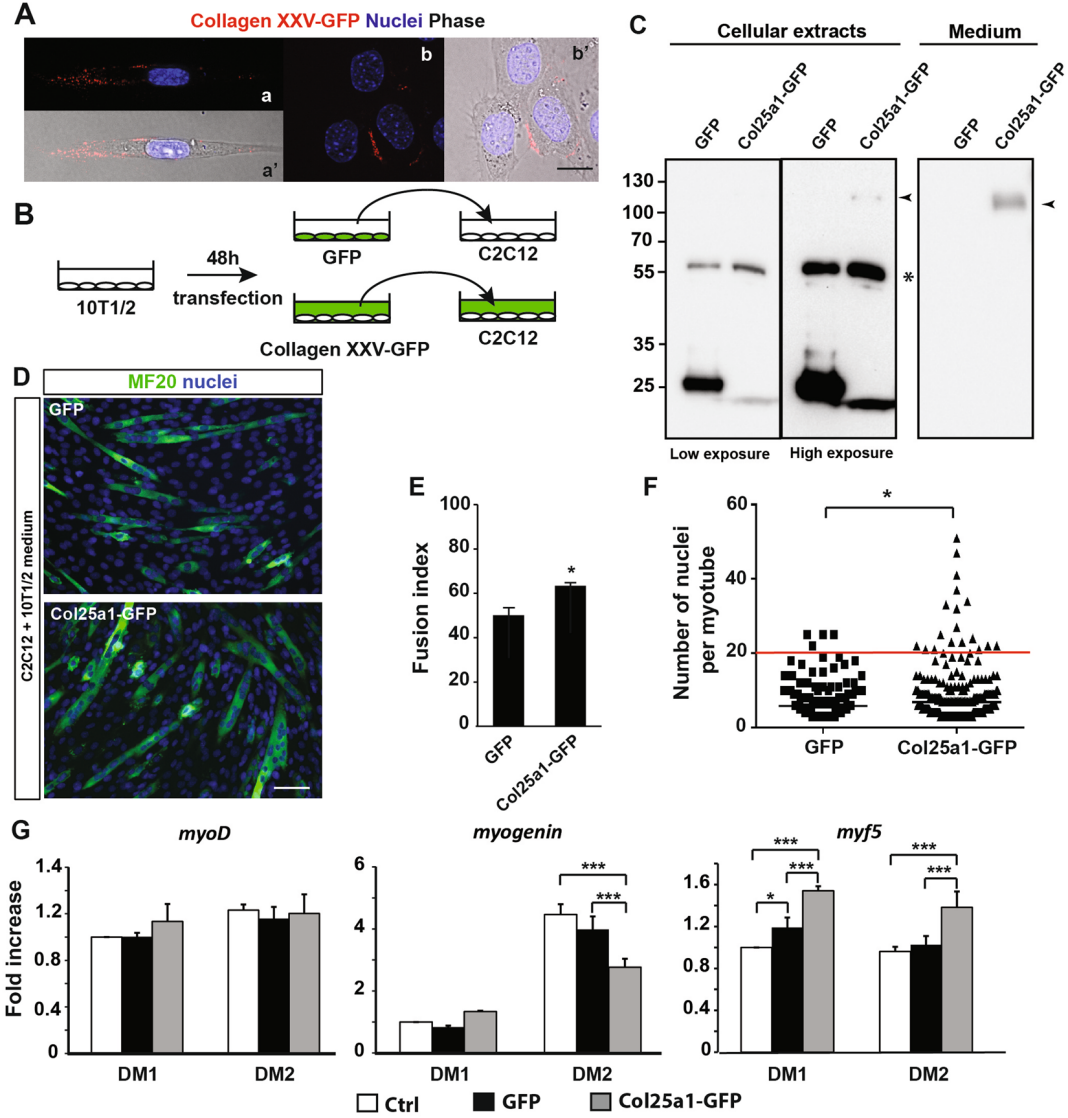
Collagen XXV containing conditioned media promote fusion of myoblasts into myotubes. (**A**) C2C12 cells were transfected 48 h in a differentiation medium, with a vector encoding collagen XXV fused to GFP (Col25a1-GFP) then live cells were stained on ice with an anti-GFP antibody. After GFP staining, cells were fixed, permeabilized and stained with Hoechst (nuclei in blue). a’ and b’ correspond to cells in a and b respectively, with phase contrast microscopy. Scale bar: 15 µm. (**B**) Strategy used to culture C2C12 cells in differentiating medium in the presence (Collagen XXV-GFP) or absence (GFP) of the soluble ectodomain of collagen XXV. (**C**) Immunoblot analyses of 10T1/2 cells transiently transfected 48 h with a vector encoding GFP (GFP) or Col25a1-GFP. Left panel: cell lysate; right panel: culture media. Note that Collagen XXV-GFP fusion proteins (arrowhead) are abundant in the appropriate culture media and barely detectable in the cell lysate. The same gel presenting the different cell lysates is presented twice, one corresponding to a low exposure (left), the other one a high exposure (right). 20 µg proteins from cellular extracts and 20 µg proteins from each culture medium were loaded. * non specific band. (**D**) Representative immunofluorescence images of C2C12 cells cultured four days in a low serum medium of transfected 10T1/2 cells, as described in B. Differentiating myoblasts and myotubes were marked by the MF20 antibody (green). Total nuclei were stained with Hoechst. Scale bar: 100 µm. (**E**) Fusion index of myoblasts in D, calculated as percentage nuclei in MyHC positive myotubes (≥3 nuclei) of total nuclei. (**F**) Distribution of myotubes with the corresponding number of nuclei in each culture condition described in B. n = 3 different experiments. *P < 0.05 vs GFP. (**G**) Expression of *myod*, *myogenin* and *myf5* was analyzed by RT-qPCR in cells cultured 1 (DM1) or 2 days (DM2) in differentiation 10T1/2 conditioned media, as described in B or in classic differentiation medium (Ctrl). Data are means ± s.e.m of three different experiments. *P < 0.05; ***P < 0.001.

Once synthesized, collagen XXV is targeted at the plasma membrane where the extracellular C-terminal domain could be cleaved by furin^10^. To test the localization of collagen XXV in muscle cells, we transfected C2C12 myoblasts with expression vectors encoding either GFP alone (GFP) or collagen XXV fused to GFP at its C-terminal end (Collagen XXV-GFP) (Fig. 3A). Collagen XXV-GFP was detected on the surface of myoblasts, by staining live cells with an anti-GFP antibody, a method used to detect plasma membrane proteins^17^ (Fig. 3Aa,Aa’). We observed that collagen XXV-GFP was concentrated on a restricted region of the cell surface in some myoblasts (Fig. 3Ab,Ab’). To further characterize the role of collagen XXV during muscle differentiation, 10T1/2 fibroblasts were transfected with expression vectors encoding either GFP alone or collagen XXV-GFP (Fig. 3B). 48 h after transfection, the collagen XXV-GFP fusion protein was barely detectable in the cellular extracts, whereas the control GFP was highly expressed in the control transfected cells (Fig. 3C). These results suggest that the ectodomain of collagen XXV fused to GFP might be cleaved by furin and liberated from the membrane as a secreted form in transfected 10T1/2 cells, as detected in the culture media of Col25a1-GFP transfected cells (Fig. 3C). To determine whether the ectodomain of collagen XXV is able to regulate myogenic differentiation, differentiating C2C12 myoblasts were cultured four days with the culture medium of GFP or Col25a1-GFP transfected 10T1/2 fibroblasts (Fig. 3B,D). Fusion index was significantly higher when C2C12 were differentiating in the presence of the ectodomain of collagen XXV in the medium (Fig. 3E). We observed a significant increase in large myotubes with more than 20 nuclei, as cells were cultured with the culture medium of Col25a1-GFP transfected 10T1/2 cells compared to the myotubes monitored in control conditions (Fig. 3D,F). To test whether the ectodomain of collagen XXV activates the differentiation program of C2C12 cells, we analyzed by RT-qPCR the expression of MyoD, Myf5 and myogenin in C2C12 cells cultured 1 or 2 days in the conditioned media from 10T1/2 cells previously transfected either with GFP or Col25a1-GFP (Fig. 3G). Although the amount of *myod* transcripts was similar in each condition tested, the expression of the pro-differentiation myogenic factor *myogenin* was down-regulated in C2C12 cells cultured 2 days in the conditioned medium of Col25a1-GFP transfected 10T1/2 cells, compared to C2C12 cells cultured 2 days in classic differentiation medium or in conditioned media from GFP transfected 10T1/2 cells. This result suggests that the presence of the ectodomain of collagen XXV in the culture medium likely inhibits the differentiation program of C2C12 cells. In contrast to *myogenin*, *Myf5* is highly expressed in proliferating myoblasts and its expression decreases as myoblasts differentiate^18^. We observed a significant increase of *myf5* transcript levels in C2C12 cells cultured 1 or 2 days in the presence of collagen XXV-GFP in the differentiation medium compared to control media or conditioned media from GFP transfected 10T1/2 cells (Fig. 3G). Altogether, these results indicate that conditioned media from col25a1-GFP transfected 10T1/2 cells promote fusion of C2C12 cells, without the activation of differentiation.

**Figure 4:**
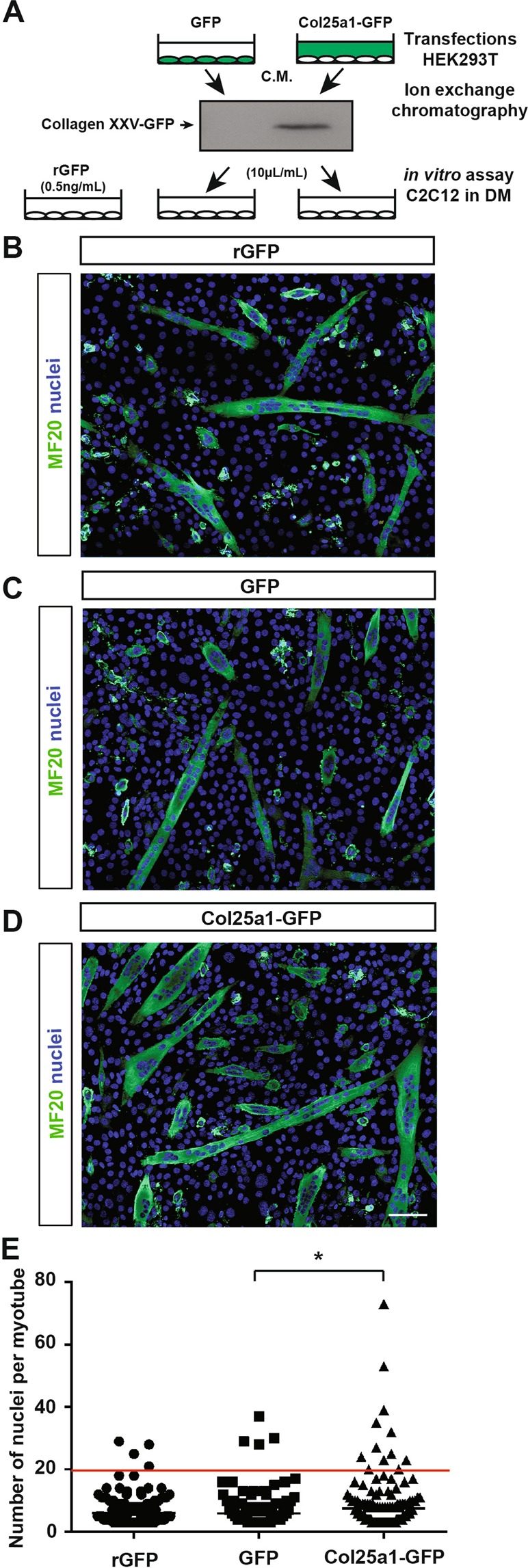
Enriched collagen XXV in the culture medium promotes the formation of myotubes with increased number of nuclei. (**A**) HEK293T cells were transiently transfected with a vector encoding collagen XXV fused to GFP (Col25a1-GFP) or GFP alone (GFP). Ion exchange chromatography of conditioned media (C.M.) was used to enrich Collagen XXV-GFP released in the former condition as assessed by Western blotting. The corresponding fractions obtained after chromatography of the GFP C.M. were pooled to be used as negative control in functional assays. Next, C2C12 cells were treated with 10 µL/mL of the fractions described above or with 0.5 ng/mL recombinant GFP (rGFP). (**B**–**D**) Representative immunofluorescence images of C2C12 cells treated four days in a low serum medium as described in A. Differentiating myoblasts and myotubes were marked by the MF20 antibody (green). Total nuclei were stained with Hoechst. Scale bar: 100 µm. (**E**) Distribution of myotubes with the corresponding number of nuclei in each culture condition described in A. *P < 0.05.

As conditioned media from 10T1/2 cells might contain many factors which could interfere with differentiation and fusion of C2C12 cells, we next tried to confirm the pro-fusion effect of the collagen XXV-GFP on differentiating C2C12 cells, by minimizing the action of these factors. To do so, we used HEK293T cells to get the highest rate as possible of collagen XXV-GFP proteins, after 48 h transfection. We then enriched collagen XXV-GFP from conditioned media of transfected HEK293T cells by ion exchange chromatography (Fig. 4A, Sup. Fig. 1). We identified and pooled the fractions the most enriched in collagen XXV-GFP. As negative control, we pooled the corresponding fractions eluted from the conditioned media of GFP transfected HEK293T cells (Fig. 4A, Sup. Fig. 1). We observed a significant increase in the number of myotubes containing more than 20 nuclei when C2C12 cells were treated with collagen XXV-GFP enriched fractions compared to myotubes monitored in cultures treated with the control fractions or with recombinant GFP (rGFP) (Fig. 4B–E). These results strengthen those obtained with differentiating C2C12 cultured in conditioned media from the different transfected 10T1/2 cells, indicating that the soluble collagen XXV ectodomain is sufficient to promote the fusion of myoblasts into myotubes. Combined with our *in vivo* data, these results demonstrate that collagen XXV is required for muscle formation during mouse development, in regulating fusion of myoblasts into multinucleated myofibers.

### Post-transcriptional regulation of Col25a1

**Figure 5:**
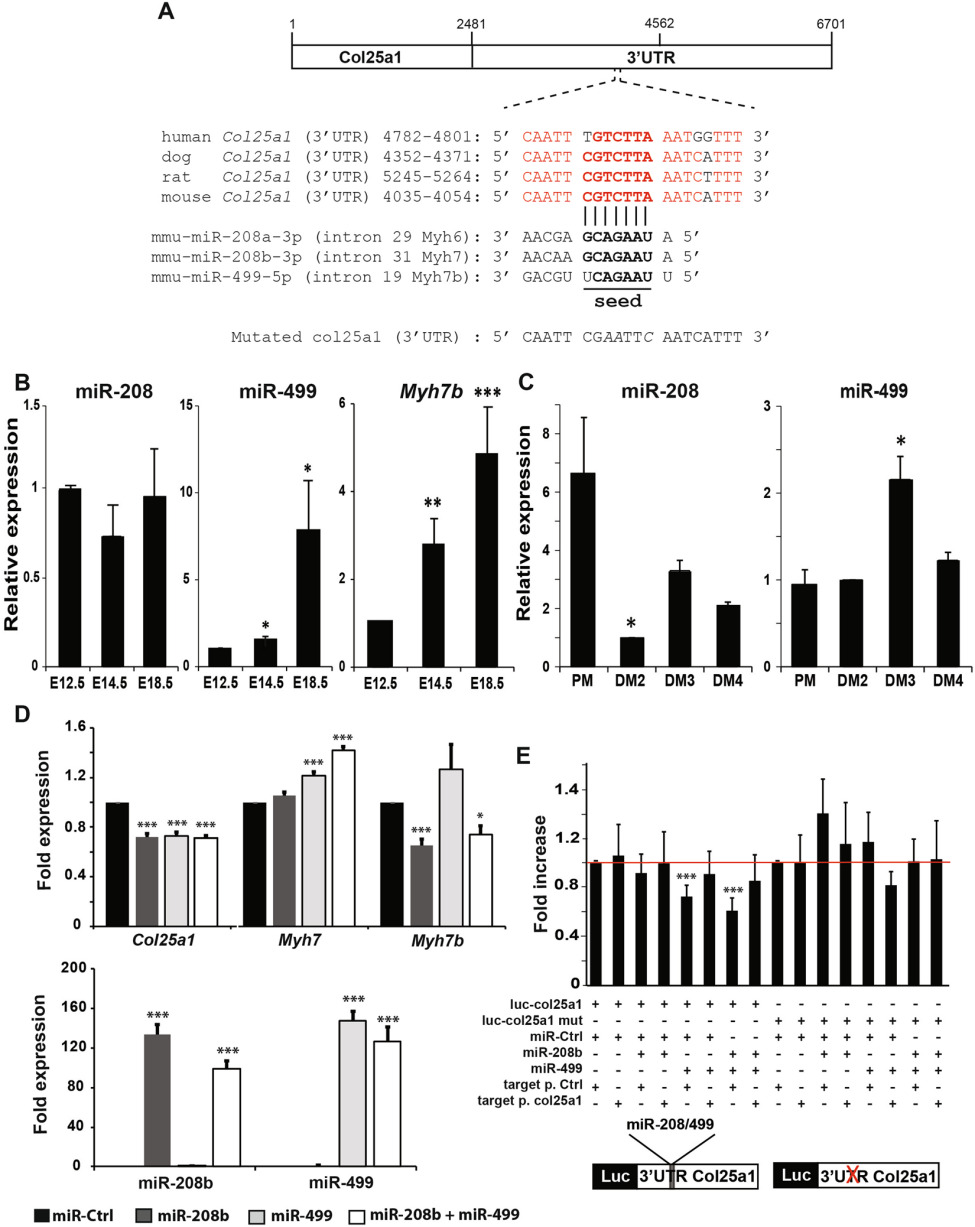
*Col25a1* transcripts are regulated by miR-499 in skeletal muscles. (**A**) Sequence alignment show the target site of miR-208a, miR-208b and miR-499 in the 3′UTR of mouse *Col25a1*, as predicted by miRDB. This site is highly conserved between human, dog, rat and mouse. A mutation in the seed region is indicated. (**B**) The expression of miR-208, miR-499 and *Myh7b* was analyzed by RT-qPCR from E12.5, E14.5 WT limb buds and E18.5 WT hindlimb muscles. Data are means ± s.e.m of three independent experiments. *P < 0.05; **P < 0.01; ***P < 0.001 vs E12.5. Two to six embryos were analyzed in each experiment. (**C**) Juvenile satellite cells were isolated from WT 10 day old mice and expanded. The expression profile of miR-208 and miR-499 was analyzed by RT-qPCR in cells cultured in proliferation medium (PM), or 2 (DM2), 3 (DM3) or 4 (DM4) days in differentiation medium. Data are means ± s.e.m of three different experiments. *P < 0.05 vs PM. (**D**) Expanded WT myoblasts were transfected in differentiation medium with 10 nM miRNA mimics (miR-Control, miR-208b and/or miR-499). miR-208b, miR-499, *Col25a1*, *Myh7* and *Myh7b* mRNA levels were quantified by RT-qPCR 48 h after transfections. The values are means ± s.e.m of three different experiments, each performed in duplicate. *P < 0.05; ***P < 0.001 vs miR-Ctrl. (**E**) miR-499 directly represses *Col25a1* 3′UTR in luciferase assays in 10T1/2 fibroblasts, in a specific manner, since a specific miScript Target Protector (target p.) for the miR-208b/miR-499 binding site on the 3′UTR of the *Col25a1* gene rescued the inhibition. The repression is abolished by mutation of the miR-499 binding site in the *Col25a1* 3′UTR. The values are means ± s.e.m. from three to six independent experiments, each performed in duplicate. Values in graphs represent means ± s.e.m. ***P < 0.001 vs miR-Ctrl + target p. Ctrl.

Gene expression is regulated at the transcriptional level but also post-transcriptionally by classes of non-coding RNAs^19^. As *Col25a1* expression is transient *in vitro* and *in vivo* (Fig. 2), we screened for microRNA binding sites within the 3′UTR of the *Col25a1* gene using the miRDB program (http://mirdb.org/). We identified miR-208a (mmu-miR-208a-3p) and miR-208b (mmu-miR-208b-3p) as putative candidates to repress *Col25a1* in young myotubes, on a seed site highly conserved in mammals (Fig. 5A). miR-208a and miR-208b are both intronic and encoded by two distinct slow myosin genes, *Myh6* and *Myh7* respectively^20^. This family of miRNAs has a third member, miR-499 (mmu-miR-499-5p), with a sequence highly related to those of miR-208a and miR-208b (Fig. 5A). miR-499 is also intronic, since it is encoded by intron 19 of the slow *Myh7b* gene. As *Myh6*/*miR-208a* is exclusively expressed in the heart^21^, we focused our analyses on *Myh7/miR-208b* and *Myh7b/miR-499*, even if miR-499 was not predicted to target the 3′UTR of the *Col25a1* transcripts (Fig. 5A). To test whether these miRNAs could repress *Col25a1* expression, we analyzed by RT-qPCR the expression of miR-208b and miR-499 and their host genes, during mouse embryogenesis (Figs 2A, 5B) and myogenic differentiation (Fig. 5C). The sequences of *miR-208a* and *miR-208b* are so close to each other that it is impossible to discriminate them by RT-qPCR. However, we assumed that the miR-208 transcripts detected here were miR-208b transcripts, since *miR-208a* is specifically expressed in the heart. During embryonic myogenesis, miR-208b levels were relatively similar in limb muscles (Fig. 5B), despite a net increase of the transcripts of its host gene (Fig. 2A). In contrast to miR-208b, miR-499 transcript levels exhibited a reverse expression profile to that of *Col25a1*, like its host gene, *Myh7b* (Figs 2A, 5B). miR-499 expression was indeed about eight fold higher in limb muscles of E18.5 WT embryos compared to limbs from E12.5 or E14.5 WT embryos, suggesting that miR-499 is a better candidate than miR-208b to repress *Col25a1* expression during muscle development. Next, we analyzed the expression profile of miR-208b and miR-499 during differentiation of primary muscle cells (Fig. 5C). Both miRNA expression was low 2 days after differentiation, time point when *Col25a1* transcripts were the most abundant. As *Col25a1* transcripts decreased (from day 3 of differentiation), miR-208 and miR-499 levels increased, suggesting that these two miRNAs could target *Col25a1* transcripts during myogenic differentiation in culture.

To more directly establish the regulation of *Col25a1* by miR-208b and miR-499, we manipulated miR-208b and miR-499 in cultured differentiating myoblasts. Transient transfections of synthetic miR-208b, miR-499 or both decreased significantly endogenous *Col25a1* transcripts of about 30%, in differentiating primary myoblasts after 48 h transfection and differentiation (Fig. 5D). The efficiency to repress *Col25a1* transcripts was similar for each microRNA and co-transfection of both miR-208b and miR-499 did not induce further decrease in *Col25a1* mRNA abundance, suggesting a competition between these microRNAs on the 3′UTR of *Col25a1*. In parallel, we noticed that increased levels of miR-499 specifically increased the amount of *Myh7* transcripts, while *Myh7b* expression seemed to be repressed by miR-208b (Fig. 5D), suggesting a cross-regulation of these microRNAs family members with their host genes. Next, we tested the putative miR-208b/miR-499 binding site on the 3′UTR of the *Col25a1* gene by luciferase assays. 10T1/2 fibroblasts were transiently co-transfected with miR-208b, miR-499 or both, in the presence of a miScript Target Protector specific for the miR-208b/miR-499 binding site on the 3′UTR of the *Col25a1* gene or its related negative control and a luciferase reporter fused to the 3′UTR of the *Col25a1* gene. Transient transfections of synthetic miR-499 alone or in combination with miR-208b significantly decreased the *Col25a1* 3′UTR reporter activity, and this repression was abolished in the presence of the miScript target protector specific to the miR-208b/miR-499 binding site of the 3′UTR of the *col25a1* gene (Fig. 5E). In contrast to miR-499, transfection of synthetic miR-208b alone did not significantly modify the luciferase activity of the reporter, suggesting that miR-499 is probably the main regulator of *col25a1* transcripts (Fig. 5E). These repressions were completely abrogated when miR-208b and/or miR-499 were co-transfected with a luciferase reporter construct harboring a mutated miR-208b/miR-499 binding site in the *Col25a1* 3′UTR. These results provide evidence for a consistent post-transcriptional regulation of *Col25a1* transcripts mainly by miR-499, during muscle differentiation and development.

## Discussion

### Implication of the extracellular matrix in primary myogenesis

The ECM has long been known to be important for muscle integrity and function. In myogenesis, cell-ECM interactions contribute to the formation of myotubes in the direction and organization of muscles to be. In the adult, myofibers are surrounded by an ECM material, the basement membrane. The inner layer of the basement membrane of degenerated fibers, the basal lamina, has recently been shown to play a critical role in muscle regeneration, since it serves as a scaffold directing myogenic progenitors alignment for division, migration and therefore fusion into regenerating myotubes^22^. The basement membrane appears only 2–3 days before birth in mice. Building the muscle tissue in the embryo also need a scaffold. During fetal development, secondary myoblasts adhere to primary myotubes and use them as a scaffold for their alignment and fusion into fetal myofibers^23^. So, primary myofibers serve as a scaffold for the remaining stages of myogenesis and set the orientation of all muscles. In contrast to secondary myofibers, primary myoblasts fuse in an environment composed of an interstitial ECM. Cells interact with the ECM via a variety of receptors, the integrins being the most common. Several studies showed that β1 integrins are essential for primary myoblasts to interact with their surrounding ECM and properly fuse during embryonic myogenesis^24–26^. However, it is not clear which ligands in the ECM could be involved in these cell-ECM interactions. Here, we provide evidence that collagen XXV is part of the ECM involved in the proper set up of primary myofibers, since the genetic ablation of collagen XXV leads to the formation of short and misaligned myofibers in E12.5 and E14.5 limbs (Fig. 1). Indeed, collagen XXV expression is high during primary myogenesis and reduces as secondary myogenesis occurs (Fig. 2), suggesting an important role for collagen XXV in the early stages of the construction of the muscle tissue. Whether this collagen is able to bind β1 integrins during primary myogenesis remains to be determined. As the mouse model used in our study is a whole body knock out model, we cannot exclude that non muscle cells might express collagen XXV within the embryonic limb. However, our *in vitro* data clearly show that primary myoblasts express *Col25a1* transcripts as they proliferate and differentiate (Fig. 2). Furthermore, *Col25a1* transcripts were not detected in fibroblast-like cells isolated from limb skeletal muscles of newborn mice (data not shown). Therefore, we hypothesize that primary myoblasts might be the main source of collagen XXV within the limb bud. Overall, our data combined with studies from the literature point out the crucial role of ECM in the establishment of primary myotubes, which will be used as the scaffold for all subsequent stages of myogenesis.

### Role of collagen XXV in the fusion of myoblasts

Skeletal muscle development requires migration of muscle progenitor cells, their differentiation and fusion into myotubes before maturating. Fusion of muscle cells is probably the most poorly understood process in this mechanism, particularly in mammals. Some transmembrane molecules involved in the myoblast fusion process have recently been identified. Myomaker, a muscle specific transmembrane protein, is necessary for myoblast fusion during skeletal muscle development and regeneration^27^. To stimulate its fusogenic activity, it interacts with the transmembrane micropeptide Myomixer (also named Myomerger or Minion)^28^. This micropeptide also promotes myoblast fusion and skeletal muscle formation during development^28–30^. How this small peptide induces membrane fusion is not understood yet. Before fusion, the cells might recognize each other and adhere^31^. Moreover, during myotube formation, the negatively charged phospholipid, phosphatidylserine (PS), transiently translocates from the inner layer of the plasma membrane to the outer leaflet^32^. Recognition of cell surface-exposed PS by PS receptors, such as stabilin-2, also promotes fusion with neighboring myoblasts and skeletal muscle regeneration^33^. In addition, the externalization of PS controls the orientation of the fusion, thereby preventing random fusion and leading to the proper elongation of myotubes^34^. In this context, we identified a completely new regulator of fusion of myoblasts. In absence of collagen XXV, myoblast fusion is impaired *in vivo* at E12.5 and E14.5 (Fig. 1), whereas primary cells collected from E18.5 *col25a1*−/− mice could spontaneously fuse into myotubes as efficiently as WT ones (data not shown). Altogether, these results suggest that this collagen is rather involved in the fusion of myoblasts into myotubes during primary myogenesis, its implication during secondary myogenesis being not essential. In culture, increased levels of the soluble ectodomain of collagen XXV promotes fusion of myoblasts into multinucleated myotubes, in a manner independent from the differentiation process, since the differentiation marker *myogenin* was not increased by the soluble collagen XXV in the culture medium (Figs 3 and 4). Altogether, our data further point out the role of membrane-associated molecules in myoblast fusion, mainly during primary myogenesis. As collagen XXV is initially a transmembrane protein before being cleaved by furin (Fig. 3A), we tested whether overexpression of collagen XXV would promote fusion of 10T1/2 cells with C2C12 cells (data not shown). We did not observe any sign of heterologous fusion, indicating that collagen XXV is probably not a fusogenic protein, but rather involved in the recognition or adherence of fusion competent myoblasts. The precise mechanism by which collagen XXV is involved in the fusion process remains to be determined.

### miR-499, the first microRNA involved in myogenic fusion

In this study, we also described a new type of regulators of myogenic fusion. Indeed, by regulating the expression of *Col25a1*, miR-499 also participates to the fusion process. In contrast to miR-208b, miR499 expression is significantly increasing during embryonic myogenesis, as *Col25a1* transcripts decrease (Fig. 5B). Although it was the only one from this family not to be identified in the miRDB database as targeting mouse *Col25a1* expression, miR-499 is probably the microRNA most implicated in the *Col25a1* repression during embryonic myogenesis (our results). Furthermore, miR-499 is identified in the database as a putative regulator of human *Col25a1* transcripts. Interestingly, the expression of miR-499 and its host gene, *Myh7b*, is under the control of miR-208a in the heart^20^. This is not the case with miR-208b and *Myh7b/miR499* in the skeletal muscle tissue, since overexpression of miR-208b in differentiating primary myoblasts decreased the levels of *Myh7b* transcripts, whereas *miR-499* levels were not reduced (Fig. 5D). According to the miRDB database, the murine *Myh7b* gene would be targeted at its 3′ UTR by only three microRNAs on seed regions completely different from the miR-208b one. Therefore, our results rather suggest that one of the targets of miR-208b could be the *Myh7b* pre-mRNA itself. One can hypothesize that miR-208b targets the miR-499 sequence in intron 19 to regulate transcription and/or splicing of *Myh7b*, inducing downregulation of its expression. Indeed, it has been described that a specific alternative splicing of *Myh7* transcripts blocks *Myh7* translation and activates *Myh7b* mRNA degradation^35^. Moreover, our results indicate that miR-499 modulates *Myh7* expression. Since overexpression of miR-499 induces *Myh7* overexpression and not a downregulation, miR-499 could stabilize *Myh7* transcripts (Fig. 5D). In total, these results point out an original cross-regulation of miR-499 and miR-208b on the expression of their host genes, *Myh7* and *Myh7b* respectively. Similar to these specific functions on *Myh7* and *Myh7b* expression, these microRNAs seem to have distinct mRNA targets, *Col25a1* transcripts being a favorite target for miR-499, even if miR-208b overexpression could also repress *Col25a1* expression in cultured differentiating primary myoblasts (Fig. 5D,E). Our results suggest that miR-499 transiently participates *in vitro* and *in vivo* to myogenic differentiation by controlling myoblasts fusion as myotubes mature and activates the expression of *Myh7* and *Myh7b*. Thereby, this microRNA would control the amount of nuclei within each myotube by repressing expression of collagen XXV. Whether this microRNA could control expression of other transcripts involved in myoblast fusion remains to be determined.

Fusion of myoblasts during embryonic development, as well as in adulthood, appears to be a critical process, which must be highly and tightly controlled by several mechanisms. Here, we show for the first time the implication of a collagen in this process. This transmembrane collagen is transiently expressed and tightly regulated by a muscle specific microRNA, giving the first evidence of the involvement of microRNAs in this cellular mechanism.

## Methods

All methods were carried out in accordance with approved biosafety and radiation guidelines for Université Paris Descartes and adhered to European and French Guidelines. All animal experiments were performed following protocols approved by the local animal protection committee (Comité d’Ethique en matière d’Expérimentation Animale Paris Descartes - CEEA 34). All animal procedures adhered to the guidelines of the French Ministry of Education, Research and Innovation, in accordance with the European Communities for Experimental animal use (2010/63/EU).

### Animals

Institutional animal care and use committee at Université Paris Descartes, [Laboratoire d’Expérimentation animale et transgenèse (LEAT) SFR Necker Broussais (A 75-14-08)], has approved protocols in accordance with the national authority (Ministère de l’Enseignement Supérieur, de la Recherche et de l′Innovation, France) guidelines based on European Union Directive 2010/63/EU. *Col25a1* null mice were described previously^7^.

### Cell culture

Limb muscles from P10-P14 wild-type mice were dissected and subjected to a gentle collagenase (Sigma) and dispase (Gibco) digestion. The cell suspension was filtered (40 µm pore size) and incubated with the following antibodies: anti-CD45 (1/60, Clinisciences), anti-TER119 (1/60, Becton-dikinson), anti-Sca1 (1/25, Becton-dikinson), anti-CD34 (1/40, Becton-dikinson), anti-α7-integrin (1/30, R&D). CD34/α7-integrin double positive cells were positively selected using a FACSAria (Becton-dikinson), while cells positive for CD31, CD45 and Sca1 were negatively selected. Sorted muscle progenitor cells were plated on gelatin-coated dishes and cultured in DMEM (Dulbecco’s modified Eagle’s medium)/F-12 (Gibco) containing 20% FBS (Gibco), 1% Penicillin–Streptomycin (Gibco) and 2% Ultroser (Pall). Myogenic differentiation was induced by shifting the proliferation medium to differentiation medium (DM): DMEM/F-12(Gibco), containing 2% horse serum (Gibco) and 1% Penicillin–Streptomycin.

C2C12, 10T1/2 and HEK293T cells were cultured in high glucose DMEM (Gibco) supplemented with 20% (for C2C12 cells) or 10% (for 10T1/2 and HEK293T cells) fetal bovine serum (FBS) (Gibco) and 1% penicillin/streptomycin (Gibco) at 37 °C under 5% CO_2_.

### Cloning

The *Col25a1* expressing construct was generated from a mouse cDNA library. The *Col25a1* sequence was cloned in frame into the pCI neo-EGFP reporter vector, using the following primer sequences:

Forward 5′- ATAGGCTAGCCTCGAATGTTGGTGAAGAAGCTTGC-3′;

Reverse 5′- TGGTGGCGACCGGTGGATCCCGGGACTTTTGCCAGCAGCC-3′.

The luc-Col25a1 vector contains 4 kb of the 3′UTR sequence of the *Col25a1* transcript, cloned into the pGl 4.23 reporter vector at the Xho I site. The putative miR-499/miR208b target site was mutated in the 3′UTR of the *Col25a1* gene by directed mutagenesis, using the following primers:

Forward 5′- CTCAATATACGAAACAATTCGAATTCAATCATTTGGGGGAC-3′,

Reverse 5′ GTCCCCCAAATGATTGAATTCGAATTGTTTCGTATATTGAG-3′.

The integrity of all constructs was confirmed by sequencing.

### Cell transfection and luciferase reporter assays

Primary myoblasts were transfected 48 h with 10 nM of miRIDIAN miRNA mimics for mmu-miR-208b-3p or mmu-miR-499-5p or negative control (miR-Ctrl) (Dharmacon) using Dharmafect 3 reagent (Dharmacon). For transient transfections, 10T1/2 cells were seeded 24 h on 24 well plates before transfections at 50 to 60% confluence. For luciferase reporter assays, 10T1/2 cells were co-transfected with 4 nM of miRNA mimics or negative control (miR-Ctrl) (Qiagen), 4 nM miScript col25a1 target protector or negative control (Qiagen) and 0.1 µg/well of luc-Col25a1 or mutated luc-Col25a1 plasmids using Dharmafect Duo. 0.1 µg/well of pCMV-Renilla plasmid were included as an internal control in all transfection assays. Cells were harvested and lysed 48 h after transfection and luciferase activity was analyzed using the Dual Luciferase Reporter Assay System (Promega).

### Conditioned media and *in vitro* differentiation assay

10T1/2 cells were transfected 6 h with 1 µg GFP or Col25a1-GFP expression vectors using 10 mM PEI (Polyethylenimine) reagent. The next day, differentiation medium (DMEM high glucose, with 2% horse serum) was added to the cells (conditioned differentiation medium). In parallel, C2C12 cells were seeded at 2500 cells/cm^2^ and cultured in proliferation medium (DMEM high glucose, with 20% FBS). The day later, the conditioned differentiation medium from transfected 10T1/2 cells was added to C2C12 cells (differentiation condition day 0). Two days later, the conditioned differentiation medium of C2C12 was replaced by new conditioned differentiation medium from freshly transfected 10T1/2 cells (differentiation condition day 2). C2C12 cells were fixed at differentiation condition day 4.

### Enrichment of Collagen XXV-GFP soluble fractions and *in vitro* differentiation assay

HEK293T cells were transiently transfected 8 h with plasmids encoding the Collagen XXV-GFP fusion protein or GFP alone using lipofectamine 2000 (Invitrogen). The next day, the culture medium was shifted to serum-free culture medium containing 0.02% BSA. For both conditions, conditioned media were collected 24 h later, adjusted to 50 mM Tris-HCl pH8 then loaded onto a 1 mL HiTrapQ anion-exchange column (GE Healthcare) equilibrated in the same buffer. Bound proteins were eluted in 0.5 mL fractions along a NaCl gradient (0–500 mM). All fractions were analyzed by immunoblot to identify those containing the Collagen XXV-GFP fusion protein. The 3 fractions containing the highest amounts of Collagen XXV-GFP fusion protein were pooled and frozen until used for *in vitro* differentiation assays. The corresponding fractions eluted from the GFP-alone conditioned media were processed identically and used as negative control. For the differentiation assay, C2C12 cells were seeded at 2500 cells/cm^2^ and cultured in proliferation medium (DMEM high glucose, with 20% FBS). The next day, C2C12 cell medium was switched to differentiation medium, including 10 µL/mL of pooled fractions enriched in Collagen XXV-GFP or the same amount of the corresponding fractions from GFP-alone conditioned media, or 0.5 ng/mL of recombinant GFP (Novus biologicals). Half of the differentiation medium was refreshed 2 days later, prior fixation 4 days after the beginning of the treatments.

### RNA extraction and quantitative RT-PCR

Total RNA from tissues or cultured cells was extracted using the TRIzol reagent (Invitrogen), followed by a RQ1 RNase-free DNase treatment (Promega). Reverse transcription was realized from 1 µg total RNA using the miScript II RT kit (Qiagen) according to the manufacturer’s protocol. Quantitative real-time PCR were performed in triplicate using SYBR green Supermix (BioRad) with a CFX™ 384 Touch qPCR system (BioRad). 26S was used as a normalizer. Primers used are listed in Table 1.

**Table 1:** Primers used in qPCR.

	Sequence (5′-3′) Forward primer	Sequence (5′-3′) Reverse primer
26S	AGGAGAAACAACGGTCGTGCCAAAA	GCGCAAGCAGGTCTGAATCGTG
miR-208	ATAAGACGAACAAAAGGTTTGT	
miR-499	TTAAGACTTGCAGTGATGTTT	
Col25a1	GAAAGTGGAGCGTCTCTTGG	TGACCAGGAGGACCAGATTC
Myf5	AGGAAAAGAAGCCCTGAAGC	GCAAAAAGAACAGGCAGAGG
MyoD	GGCTCTCTCTGCTCCTTTGA	AGTAGGGAAGTGTGCGTGCT
myogenin	TGACCCTACAGACGCCCACAATC	CACACCCAGCCTGACAGACAATC
Myh3	GCAAAGACCCGTGACTTCACCTCTAG	GCATGTGGAAAAGTGATACGTGG
Myh7	AGGGCGACCTCAACGAGAT	CAGCAGACTCTGGAGGCTCTT
Myh7b	CACCTTTGTGGACAGCAGAA	TAGAAGCCCAGCCTTGAAGA

### Immunocytochemistry

Cultured cells were rinsed with PBS and fixed in 2% paraformaldehyde (PFA) for 10 min at room temperature. Cells were subsequently permeabilized with 0.2% Triton X–100 for 10 minutes and incubated overnight at 4 °C with anti-MF20 (1/100, DSHB). Nuclei were stained with Hoechst (20 µg/mL, Sigma). To stain live cells, the latter were first washed with PBS and incubated 30 minutes on ice in anti-GFP antibody (Abcam, ab5450) diluted 1/800 in 3% BSA/PBS, followed by 10 minutes fixation with 4% PFA/PBS, 10 minutes permeabilization in 0.2% Triton/PBS and incubation with secondary antibody. Nuclei were then stained with Hoechst. All these cultures were visualized on a Zeiss ApoTome2 fluorescent microscope.

### Histological analysis and immunofluorescence

Trunk and forelimbs from E12.5 and E14.5 embryos were dissected, embedded in optimal cutting temperature (O.C.T.) and frozen in cold isopentane. 10 µm transverse cryostat sections were realized and fixed with acetone. After 1 h in PBS with 0.1 M glycine, sections were incubated overnight at 4 °C with the following antibodies: anti-MF20 (1/100, DSHB), anti-embryonic MyHC (1/40, Santa Cruz sc-53091). Nuclei were stained with Hoechst (20 µg/mL, Sigma). Sections were visualized on a NanoZoomer 2.0-HT slide scanner (Hamamatsu).

### Western blot analysis

Total proteins were extracted from cultured cells with protein lysis buffer (2% SDS, 50 mM Tris-HCl pH 6.8, 10% glycerol, 100 mM DTT). To analyze the presence of the fusion protein collagen XXV-GFP in the cell culture medium, transfected 10T1/2 cells were cultured overnight in a serum free medium. Cell culture media were then collected and concentrated using Amicon 10 K (Millipore). Bio-Rad protein assay with BSA standard was performed to measure protein concentration of samples. To detect collagen XXV-GFP proteins in conditioned media of transfected HEK293T cells, cells were cultured overnight in a serum free medium containing 0.02% BSA and an aliquot of the conditioned media was used for Western blotting. 20 µg proteins were separated by sodium dodecyl sulfate polyacrylamide gel 10% electrophoresis. The separated proteins were transferred to a PVDF membrane before immunoblotting with the following primary antibody: anti-GFP (1/2000, Santa Cruz sc-9996). Anti-mouse-HRP (Cell signalling 7076 S) was used as a secondary detection antibody. Finally, immunoreactive bands were detected using the ECL chemiluminescence detection.

### Statistical analysis

The fusion index was calculated as the percentage of nuclei in myosin positive myotubes with more than 3 nuclei of total nuclei. The number of nuclei in myotubes was manually counted. The results are presented as means ± s.e.m. Statistical analyses were performed using *t*-test or two-way ANOVAs followed by a two-way repeated-measures analysis of variance with a Tukey’s multiple comparisons test (Figs 3G, 4E). Statistical significance was accepted at a *P* value < 0.05.

## Supplementary information

Sup dataset 1
