# Author Correction: Collagen XXV promotes myoblast fusion during myogenic differentiation and muscle formation

**DOI:** 10.1038/s41598-019-53896-7

**Published:** 2019-11-21

**Authors:** Tristan J. M. Gonçalves, Florence Boutillon, Suzie Lefebvre, Vincent Goffin, Takeshi Iwatsubo, Tomoko Wakabayashi, Franck Oury, Anne-Sophie Armand

**Affiliations:** 1 INSERM UMRS 1124, 45 rue des Saints-Pères, F-75270 Paris cedex 06, France; 2 Institut Necker-Enfants Malades, Inserm, U1151, 14 rue Maria Helena Vieira Da Silva, CS 61431, Paris, F-75014 France; 3 Université Paris Descartes, Sorbonne Paris Cité, Paris, France; 4 Department of Neuropathology, Graduate School of Medicine, the University of Tokyo, Tokyo, 113-0033 Japan; 5 Department of Innovative Dementia Prevention, Graduate School of Medicine, the University of Tokyo, Tokyo, 113-0033 Japan

Correction to: *Scientific Reports* 10.1038/s41598-019-42296-6, published online 10 April 2019

In the original version of this Article, Takeshi Iwatsubo was incorrectly affiliated with both ‘Department of Neuropathology, Graduate School of Medicine, the University of Tokyo, Tokyo, 113-0033, Japan’ and ‘Department of Innovative Dementia Prevention, Graduate School of Medicine, the University of Tokyo, Tokyo, 113-0033, Japan’. The correct affiliation is listed below.

Department of Neuropathology, Graduate School of Medicine, the University of Tokyo, Tokyo, 113-0033, Japan.

In addition, Tomoko Wakabayashi was only affiliated with ‘Department of Neuropathology, Graduate School of Medicine, the University of Tokyo, Tokyo, 113-0033, Japan’. The correct affiliations are listed below.

Department of Neuropathology, Graduate School of Medicine, the University of Tokyo, Tokyo, 113-0033, Japan.

Department of Innovative Dementia Prevention, Graduate School of Medicine, the University of Tokyo, Tokyo, 113-0033, Japan.

These errors have now been corrected in the HTML and PDF versions of this Article.

