# Supplementary material for: Collagen XXV promotes myoblast fusion during myogenic differentiation and muscle formation
Source: Sci Rep. 2019 Apr 10;9:5878. doi: 10.1038/s41598-019-42296-6 (PMC6458142; doi:10.1038/s41598-019-42296-6)
Supplement: Supplementary file 1 — Sup dataset 1 [file 41598_2019_42296_MOESM1_ESM.pdf]

Supplementary Information

Article in *Scientific Reports*

Collagen XXV promotes myoblast fusion during myogenic differentiation and muscle formation

Tristan J.M. Gonçalves, Florence Boutillon, Suzie Lefebvre, Vincent Goffin, Takeshi Iwatsubo, Tomoko Wakabayashi, Franck Oury, Anne-Sophie Armand

Supplemental Figure 1

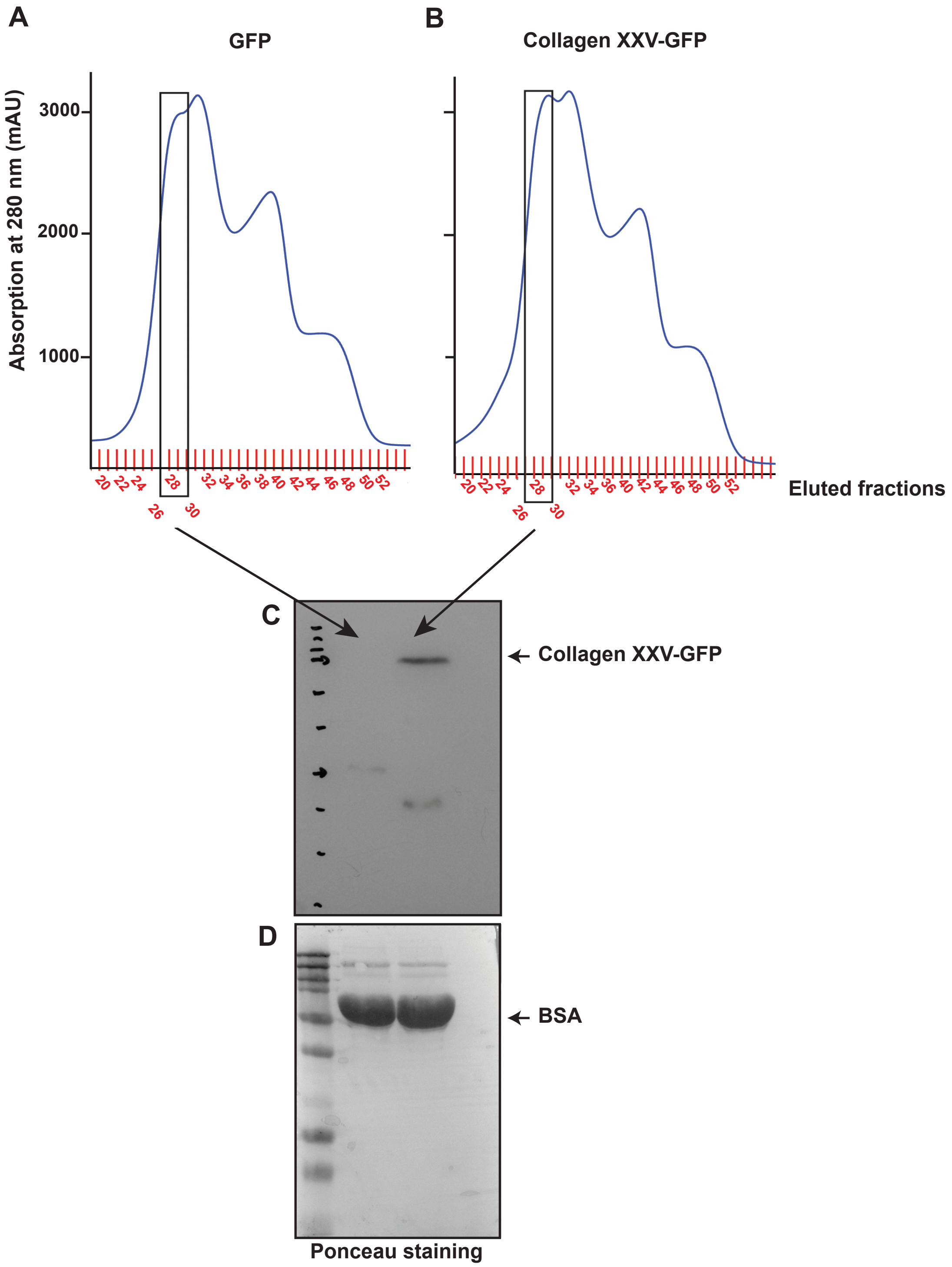

Sup. Fig. 1: Enrichment of collagen XXV-GFP by ion exchange chromatography.
Conditioned media from GFP (A) and Col25a1-GFP (B) transfected HEK293T cells were collected and fragmented by anion exchange chromatography. The fractions the most enriched in collagen XXV-GFP (#27-29, box in B) were selected and pooled. The corresponding fractions (#27-29, box in A) from ion exchange chromatography of conditioned medium of GFP-transfected HEK293T cells were also pooled to be used as negative control in C2C12 cell differentiation bioassay. The presence (or the absence) of collagen XXV-GFP in both pooled fractions was assessed by Western blot (C). Ponceau red staining of the immunoblots (D) showed that protein contaminants co-eluting with collagen XXV-GFP mainly involved BSA added in serum-free culture media of HEK293T cells.

Supplemental Figure 2

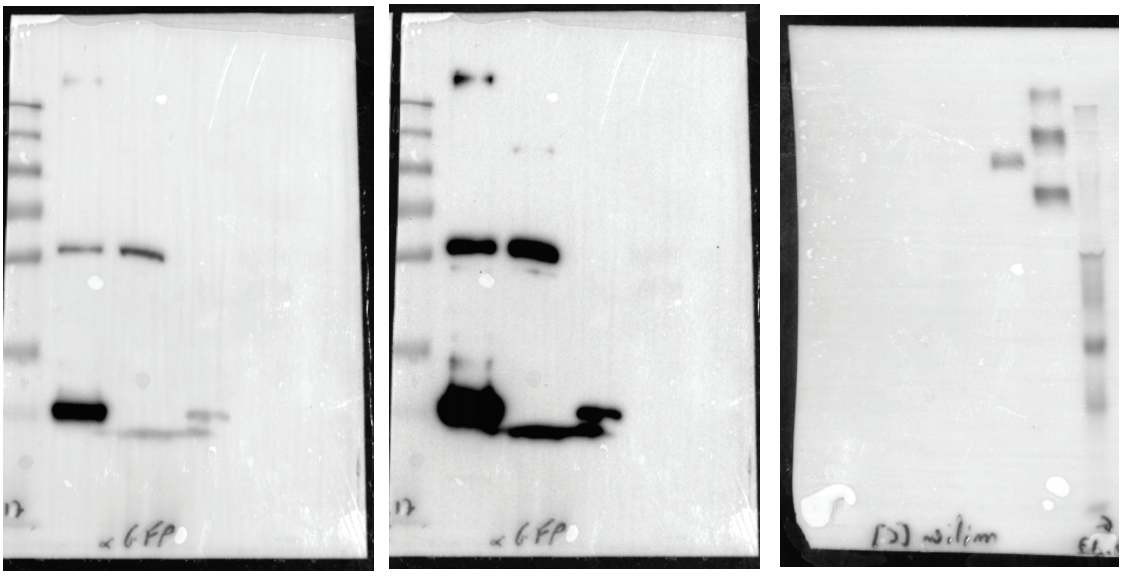

Sup Fig. 2: Full-length western blot of Figure 3C
